# RUNX2 and the PI3K/AKT axis reciprocal activation as a driving force for tumor progression

**DOI:** 10.1186/s12943-015-0404-3

**Published:** 2015-07-25

**Authors:** Karine A. Cohen-Solal, Rajeev K. Boregowda, Ahmed Lasfar

**Affiliations:** Rutgers Cancer Institute of New Jersey, Department of Medicine, Division of Medical Oncology – Rutgers, the State University of New Jersey, Robert Wood Johnson Medical School, 195 Little Albany Street, New Brunswick, New Jersey 08903 USA; Department of Pharmacology and Toxicology, Ernest Mario School of Pharmacy, Rutgers, The State University of New Jersey, Piscataway, New Jersey 08854 USA; Rutgers Cancer Institute of New Jersey, New Brunswick, New Jersey 08903 USA

**Keywords:** Transcription factor, RUNX2, Migration, Invasion, Phosphatidylinositol 3-kinase (PI3K), Protein Kinase B (PKB/AKT)

## Abstract

From the first reported role of the transcription factor RUNX2 in osteoblast and chondrocyte differentiation and migration to its involvement in promigratory/proinvasive behavior of breast, prostate, and thyroid cancer cells, osteosarcoma, or melanoma cells, RUNX2 currently emerges as a key player in metastasis. In this review, we address the interaction of RUNX2 with the PI3K/AKT signaling pathway, one of the critical axes controlling cancer growth and metastasis. AKT, either by directly phosphorylating/activating RUNX2 or phosphorylating/inactivating regulators of RUNX2 stability or activity, contributes to RUNX2 transcriptional activity. Reciprocally, the activation of the PI3K/AKT pathway by RUNX2 regulation of its different components has been described in non-transformed and transformed cells. This mutual activation in the context of cancer cells exhibiting constitutive AKT activation and high levels of RUNX2 might constitute a major driving force in tumor progression and aggressiveness.

## Introduction

The products of *RUNX* (*Runt-related transcription factor*) genes comprise a family of three closely related transcription factors, RUNX1, RUNX2 and RUNX3. These genes are defined by a highly conserved 128 amino acid DNA binding/protein-protein interaction domain, called the Runt-homology domain [1,2]. RUNX2 is a major determinant of osteoblast differentiation and regulates chondrocyte proliferation, differentiation and hypertrophy during endochondral bone formation [3–6].

The involvement of RUNX2 in tumor development, progression and metastasis is largely documented. Some evidence indicates a role for RUNX2 in T-cell lymphoma, acute myeloid leukemia and multiple myeloma [1,7–9] and bone metastasis in advanced mammary and prostate cancer [1,10–12]. However, the role of RUNX2 in promoting tumor development in mammary and prostate cancer extends beyond its pro-bone metastatic effects [10,11] and the osteolytic disease associated with these cancers [13,14]. RUNX2 regulates the expression of genes intimately associated with tumor progression, invasion and metastasis and its role in migration and invasion has been documented in different tumor cell types. RUNX2 siRNA treatment of the prostate cancer cell line PC3 decreases migration and invasion through Matrigel *in vitro* [13]. Overexpression of RUNX2 in the prostate cancer cell line C4-2B enhances its invasiveness [15]. In addition, RUNX2 overexpression upregulates transcription factors (*SOX9, SNAI2 and SMAD3*) implicated in the process of epithelial to mesenchymal transition (EMT), whose features include increased motility and invasion potential. Furthermore, RUNX2 overexpression upregulates genes involved in cellular movement and cytoskeleton remodeling [15]. High RUNX2 levels in PC3 cells are associated with development of large tumors, and increased expression of the two metalloproteinases *MMP9* and *MMP13, OPN* and *VEGF*, and secreted bone-resorbing factors (*PTHrP*, *IL-8*) promoting osteolytic disease [13]. Similarly, in human breast cancer, RUNX2 directly regulates the expression of *MMP9* and *MMP13* [16–18], *bone sialoprotein* and *OPN* [16], *IL-8* [19] and the TGFβ-induced *PTHrP* levels [14] and mediates invasion of the human breast cancer cell lines MDA-MB-231 and MCF7 [17]. Interestingly, aberrant RUNX2 expression induces EMT-like changes in normal mammary epithelial cells [20] and disrupts normal acini structure in three-dimensional cultures [19], suggesting a role for RUNX2 in promoting the early events of breast cancer progression. RUNX2 also plays a central role in mediating the pro-migratory and pro-invasive function of thyroid tumor cells, by activating the expression of *MMP2, MMP13, MMP14* and *OPN* [21]. SiRNA-mediated knockdown of RUNX2 in human colon carcinoma cells leads to decreased migration and invasion [22]. The U2OS osteosarcoma cells also demonstrate reduced motility following siRNA-mediated depletion of RUNX2. In addition, genomic promoter occupancy of RUNX2 in osteosarcoma cells identifies genes involved in motility, such as *FAK/PTK2* or *talin (TNL1)* [23]. We also demonstrated that RUNX2 knock down in melanoma cell lines significantly inhibits their migration and invasion potential [24].

In addition, the pro-angiogenic effects of RUNX2 are highly suggestive of RUNX2 as a major player in tumor promotion. These effects include endothelial cell proliferation, migration and invasion [25,26], induction of *VEGF* expression or RUNX2 physical and functional interactions with another major pro-angiogenic factor, hypoxia-inducible factor 1-a (HIF1-a) [27,28]. These studies altogether define the transcription factor RUNX2 as pro-migratory, pro-invasive and pro-angiogenic, in addition to its role in promoting the early steps of tumorigenesis in breast cancer and driving the metastatic bone disease in prostate and breast cancer. In this review, we are focusing on the functional interrelations between RUNX2 and the PI3K/AKT pathway, which contribute to cancer progression.

The oncogenic role of the PI3K/AKT axis on tumor growth extends beyond its pro-proliferative and survival effects and includes migration and invasion. One important contribution is the demonstration that PI3K function is required for TGFβ-mediated epithelial to mesenchymal transition (EMT) of the NMuMG mammary epithelial cell line. In addition, PI3K inhibition blocks both basal and TGFβ-induced cell migration of mouse breast cancer cell lines [29]. The collaboration of TGFβ autocrine signaling and the activated PI3K/AKT pathway plays a key role in cancer progression, causing the shift in TGFβ/SMAD signaling from its tumor suppressive to its tumor promoting mode [30]. As another illustration of this concept, AKT-mediated phosphorylation of the EMT transcription factor TWIST1 leads to transcriptional activation of the *TGFβ2* promoter and activated TGFβ signaling promoting EMT and breast cancer metastasis [31].

Numerous studies describe the requirement of AKT signaling for the migration and invasion of tumor cells. Overexpression of AKT or myristylated AKT (MyrAKT), which is anchored to the plasma membrane and has a constitutively active kinase activity, increases the migration and invasion of a human fibrosarcoma cell line. This study demonstrates that AKT promotes migration and invasion in a manner depending on both its membrane-translocating ability and its kinase activity. In addition, cell migration and invasion require PI3K-dependent translocation of AKT at the cell membrane, as evidenced by the inhibition of AKT translocation, cell migration and invasion by the PI3K inhibitor LY294002 [32]. The expression of subunit p110α (PI3K) siRNA or AKT1 siRNA in a human ovarian cancer cell line significantly decreases its migration and invasion [33]. CXCL12-mediated *MMP-9* expression and chemoinvasion is sensitive to PI3K inhibitors in various prostate cancer cell lines [34]. Also in prostate cancer cells, oncogenic ETS transcription factors require AKT signaling to activate a cell migration gene expression program through ETS/AP-1 binding sequences [35]. Adenoviral transfer of PTEN into melanoma cells leads to inhibition of AKT phosphorylation and suppression of melanoma cell invasion [36]. Similarly, PTEN loss increases invasion of human melanoma cells and non-transformed melanocytes, with a concomitant shift to phosphorylation of AKT2 [37]. The adaptor GAB2 involved in the activation of both RAS-ERK and PI3K/AKT signaling pathways, is overexpressed in metastatic melanoma, promoting migration and invasion of melanoma cells [38]. In addition, AKT is involved in TGFα-mediated migration of human osteosarcoma cells [39]. Therefore, the activation of AKT promotes the EMT, migration and invasion programs in a PI3K-dependent manner.

The implication of RUNX2 in signaling pathways involved in tumorigenesis, such as the transforming growth factor beta (TGFβ) signaling pathway [40,41], the WNT pathway [40] and the p53 pathway [42] has been reviewed thoroughly. Mechanistic studies mainly performed in osteoblasts and chondrocytes and in different cancer cellular systems shed light into a functional interaction and cooperation between RUNX2 and the PI3K/AKT pathway. In the context of cancer cells, this interaction might feed a positive feedback loop for the benefit of tumor progression.

## The PI3K/AKT pathway stimulates RUNX2 expression and activity through direct or indirect mechanisms (Fig. 1a).

**Fig. 1:**
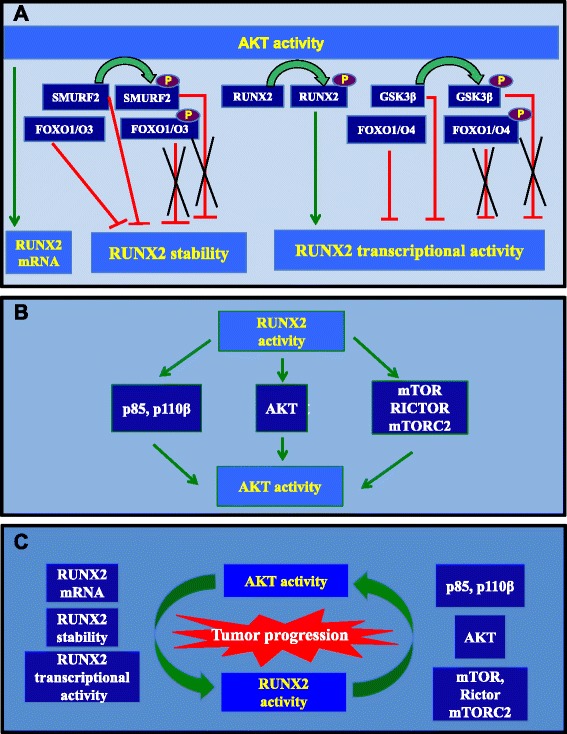
Model of RUNX2 and PI3K/AKT reciprocal interaction as a driving force for tumor progression. **a**. AKT activity results in up regulation of *RUNX2* mRNA, stabilization of RUNX2 protein or activation of RUNX2 transcriptional activity. **b**. RUNX2 transcriptional activity leads to higher expression of the PI3K subunits *p110β* and *p85*, *AKT* or *mTOR* and *RICTOR*, two major components of the mTORC2 complex. **c**. Mutual activation of AKT and RUNX2 in the context of cancer cells might favor tumor progression. A pioneering study [47] shows that RUNX2 and the PI3K/AKT axis are mutually dependent on each other in the regulation of osteoblast and chondrocyte differentiation and their migration. We propose that a similar feed-forward loop could operate in tumor cells to potentiate RUNX2 and PI3K/AKT activities and generate the pro-migratory and pro-invasive behavior of tumor cells. Studies described in paragraphs 1 and 2 support this hypothesis

### Direct regulation of the PI3K/AKT pathway on RUNX2 expression and activity

In prostate cancer cells, stable expression of constitutively activated AKT (myr-AKT) induces the expression of *RUNX2* mRNA and its target genes, such as *PIP, PGC, MMP9 and MMP13* [43]. In a model of oxidative stress-induced vascular smooth muscle cell (VSMC) calcification, H_2_O_2_ induces *RUNX2* mRNA and protein expression, resulting in VSMC calcification [44]. H_2_O_2_ also increases phosphorylated AKT levels. The H_2_O_2_-mediated *RUNX2* expression is prevented in the presence of an AKT inhibitor, suggesting that AKT-mediated induction of *RUNX2* plays a critical role in oxidative stress-induced VSMC calcification [44]. Interestingly, treatment of colorectal cancer cells with H_2_O_2_ results in a dramatic increase in total AKT and phosphorylated AKT (pAKTS473) levels [45]. Considering that RUNX2 immunoreactivity in colon carcinoma cells is associated with their aggressive clinical behavior in colon carcinoma patients [22], we propose that in a way similar to VSMC calcification, prevalent oxidative stress in cancer [46] could participate in maintaining high levels of RUNX2 through stimulation of the PI3K/AKT pathway.

In addition to inducing the expression of RUNX2, earlier experiments show that activation of the PI3K/Akt signaling pathway enhances DNA binding of Runx2 and Runx2-dependent transcription in a mouse osteoblastic cell line [47]. The promoter activity of *osteocalcin* in mouse Runx2-overexpressing osteoblastic cells is enhanced by a constitutively active form of Akt1 (Akt1^CA^) and suppressed by Akt1 siRNA. Chromatin immunoprecipitation (ChIP) assay shows that the complex between Runx2 and the *osteocalcin* promoter present in wild-type osteoblasts disappears in Akt1-/- osteoblasts [48]. In these studies, no mechanism is proposed for the PI3K/Akt-mediated increase in Runx2 DNA binding and transcriptional activity. More recently, it was shown in cell-free assays that AKT phosphorylation of RUNX2 Runt homology domain enhances its DNA binding potential on a RUNX2 consensus oligonucleotide. In addition, mutation in AKT phosphorylation sites (generated by substitution of S203/T205 (double mutant) or S203/T205/S207 (triple mutant) by Alanines) produces RUNX2 forms that have a reduced ability to bind the RUNX2 consensus oligonucleotide. Expression of the double or triple mutants of RUNX2 in breast cancer cells reduces the mRNA levels of two metastasis-related target genes, *MMP9* and *MMP13*, and reduces the RUNX2-dependent invasive potential of breast cancer cells. This study establishes RUNX2 as an AKT substrate and an important mediator of PI3K/AKT signaling in breast cancer [49].

### Indirect regulation of the PI3K/AKT pathway on RUNX2 expression and activity

In addition to transcriptional effects of the PI3K/AKT pathway on *RUNX2* expression, RUNX2 protein levels are also indirectly regulated by stabilization via AKT. In osteoblasts, RUNX2 interaction with the E3 ubiquitin ligase SMURF2 induces degradation of RUNX2 in an ubiquitin/proteasome-dependent manner [50]. During osteoblast differentiation, AKT interacts with and phosphorylates SMURF2. In addition, AKT enhances ubiquitin/proteasome-mediated degradation of SMURF2, counteracting the SMURF2-induced degradation of RUNX2. Therefore, RUNX2 increased stability by AKT is mediated by SMURF2 in osteoblasts [51]. It will be interesting to investigate whether some tumor suppressive functions of SMURF2 [52] could be related to its negative effects on RUNX2 and whether AKT-mediated degradation of SMURF2 can occur in tumors, explaining the high levels of RUNX2 in certain types of cancer [40].

A novel function of FOXO1 and FOXO3 in negatively regulating RUNX2 stability has been delineated in vascular smooth muscle cells (VSMC). FOXO1/FOXO3 knockdown inhibits RUNX2 ubiquitination, increasing RUNX2 abundance. Activation of AKT, either by PTEN deficiency or by overexpression of constitutively activated AKT, leads to nuclear exclusion of FOXO1 and FOXO3 in VSMC [53]. In the context of cancer cells, constitutive AKT activation would favor FOXO1/O3 nuclear exclusion, thereby decreasing RUNX2 ubiquitination and degradation and increasing RUNX2 stability. These studies altogether show that AKT activity positively regulates RUNX2 stability through ubiquitin/proteasome-mediated degradation of SMURF2 or nuclear exclusion of FOXO1 and FOXO3, depending on the cellular system.

Additional studies demonstrate that AKT also positively regulates RUNX2 activity through indirect mechanisms, involving FOXO1, FOXO4 and Glycogen Synthase Kinase 3β (GSK3β). The transcription factor FOXO1 physically interacts with RUNX2 in osteoblastic cells and in COS-7 cells and inhibits RUNX2 binding to its cognate site within the *osteocalcin* promoter. Upon IGF1/insulin binding to their receptors, activation of the PI3K/AKT pathway leads to phosphorylation and nuclear exclusion of FOXO1 and reactivation of RUNX2 [54]. This indirect mechanism of RUNX2 activation by the PI3K/AKT pathway is also described in human tumor cells. FOXO1 interacts with RUNX2 *in vitro* and in prostate cancer cells, and inhibits RUNX2 transcriptional activity on the *OP, IL8, VEGF and MMP13* genes. FOXO1 also inhibits RUNX2-mediated migration and invasion of prostate cancer cells [55]. The loss of PTEN in prostate cancer cells leads to activation of AKT and phosphorylation and retention of FOXO1 in the cytoplasm. The FOXO1 phosphorylation by AKT therefore abolishes FOXO1-mediated inhibition of RUNX2 and favors RUNX2-mediated gene expression, migration and invasion [55]. Interestingly, expression of PTEN and the level of FOXO1 in the nucleus inversely correlate with RUNX2 expression in prostate cancer specimens from patients with lymph nodes or bone metastasis [55]. As demonstrated in prostate cancer, FOXO4 antagonizes RUNX2 activity by physically interacting with and preventing RUNX2 to induce the expression of pro-metastatic genes, such as *PIP and PGC* [43]. As for FOXO1, AKT phosphorylation of FOXO4 results in its retention in the cytosol [56]. Therefore, in the case of hyperactive AKT signaling, the retention of FOXO4 in the cytoplasm prevents this antagonism, thereby reactivating RUNX2 and favoring expression of RUNX2-dependent metastasis genes [43]. Phosphorylation of RUNX2 at S369-S373-S377 by Glycogen Synthase Kinase 3β (GSK3β) attenuates the transcriptional activity of RUNX2, explaining the suppression of bone formation by overexpression of wild-type GSK3β or constitutively active form of GSK3β (CA-GSK3β) [57]. Since AKT phosphorylates GSK3β at S9 causing its inactivation [58], we postulate that AKT activation will result in GSK3β inactivation and restoration of RUNX2 transcriptional activity. Therefore, in tumor cells exhibiting constitutive activation of the PI3K/AKT pathway, by various mechanisms such as RAS mutation, PTEN deletion, PI3K mutation, or receptor tyrosine kinase overexpression, it is conceivable that GSK3β inactivation will prevent the repression of RUNX2 transcriptional activity.

## RUNX2 activates the PI3K/AKT pathway by regulating its components (Fig. 1b).

### Direct control of RUNX2 on the expression of the PI3K/AKT pathway components

The regulation of PI3K and Akt protein levels by RUNX2 was initially demonstrated in the context of mouse osteoblast and chondrocyte differentiation. This work shows that Runx2 upregulates PI3K subunits p85 and PIK3CB (p110β) and the kinase Akt. p110β is mainly regulated at the transcriptional level, while p85 and Akt are regulated at both transcriptional and protein levels [47]. A similar mechanism is described in the megakaryocytic leukemia (AMkL) cell line Meg-01. RUNX1 directly binds to the RUNX1 binding sites of the *PI3KCD (P110DELTA)* promoter and the *PI3KCD* gene is a direct transcriptional target of RUNX1 in the Meg-01 cells [59].

### Indirect regulation of RUNX2 on the PI3K/AKT signaling pathway

The indirect regulation of RUNX2 on the PI3K/AKT signaling pathway has been documented in prostate and breast cancer cells. In androgen-independent prostate cancer cell lines, knocking down or overexpressing RUNX2 leads to a decreased or increased level of phosphorylated AKT at serine 473 (pAKTS473) respectively by mechanisms yet to be defined [60]. A novel mechanism to activate AKT activity is demonstrated in invasive breast cancer cells. Endogenous RUNX2 is required to maintain high levels of pAKTS473 in invasive cancer cells, but not in non-invasive breast cancer cells or normal cells. RUNX2 knockdown in the invasive cancer cells results in the decrease of mRNA and proteins levels of both mTOR and RICTOR, two major components of the mTORC2 complex involved in phosphorylating AKT on serine 473. The effect on mTOR expression is mediated through direct binding of RUNX2 to the *mTOR* promoter region (-2420 to -2441) containing two highly conserved RUNX binding elements at -2420 and -2430 bp. Therefore, RUNX2 up regulates components of the mTORC2 complex, which directly phosphorylates AKT [61].

An additional illustration of an indirect regulation of RUNX2 on the PI3K/AKT pathway comes from genomic promoter occupancy of RUNX2 in osteosarcoma cells. This analysis identifies genes involved in cell adhesion and motility, such as *FAK* [23], which also signals through the PI3K/AKT pathway [62]. Therefore, in osteosarcoma cells, RUNX2 could stimulate the PI3K/AKT pathway through the positive regulation of FAK. We also demonstrated that knocking down RUNX2 in human melanoma cells results in decreased levels of the FAK protein [24] and our recent experiments show that RUNX2 regulates *FAK* mRNA (unpublished results). Therefore, also in melanoma, RUNX2 could regulate the PI3K/AKT pathway through FAK up-regulation.

## Integration of the TGFβ signaling pathway in the RUNX2/AKT feedback loop

**Fig. 2:**
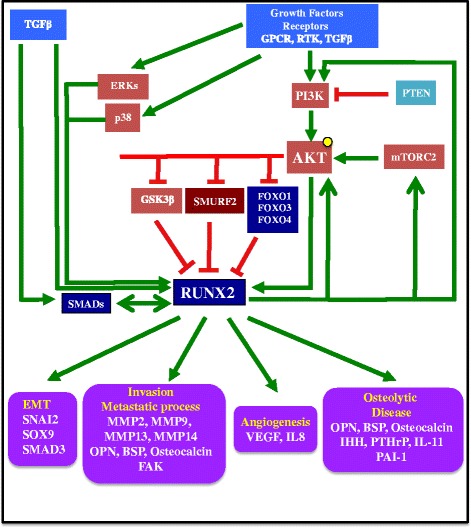
Summary of the interactions between the PI3K/AKT pathway and RUNX2 with integration of the TGFβ pathway. Some genes regulated by RUNX2 and supporting different aspects of tumor progression are represented. The activation of growth factor receptors, such as G-protein coupled receptors (GPCR), receptor tyrosine kinases (RTKs), TGFβ receptor, results in successive activation of PI3K and AKT. PTEN inactivation also results in activation of PI3K and AKT. AKT activation positively regulates RUNX2 expression and activity by direct and indirect mechanisms as shown in Fig. 1A. Reciprocally, RUNX2 activates the PI3K/AKT pathway by regulation if its components, PI3K and AKT, or regulation of mTOR and RICTOR, two majors components of mTORC2, which phosphorylates AKT on serine 473. Upon receptor activation, the kinases ERK and p38 also phosphorylate and activate RUNX2. Through partnering with SMADs transcription factors, RUNX2, (TGFβ target gene itself) activates the expression of genes involved in EMT, invasion and the metastatic cascade, angiogenesis and bone disease. OPN: osteopontin; BSP: Bone sialoprotein; MMP: Matrix metalloproteinase; FAK: Focal adhesion kinase; IHH: Indian hedgehog; PTHrP: Parathyroid hormone-related protein; VEGF: Vascular endothelial growth factor; IL8: Interleukin-8. IL11: Interleukin-11; PAI-1: Plasminogen activator inhibitor-1

In the context of cancer cells, the positive feedback loop illustrated in Figs. 1 and 2 integrates another important player in tumor progression, the TGFβ signaling pathway, in the establishment of a major process involved in migration, invasion and metastasis, the epithelial to mesenchymal transition (EMT). The role of TGFβ in cancer-associated EMT is largely documented [63]. In TGFβ-induced EMT, the downstream effectors SMADs (SMAD2 and SMAD3) stimulate gene reprogramming, by activating directly or indirectly the expression of the master EMT transcription factors *SNAI1, SNAI2/SLUG, ZEB1, ZEB2 and TWIST1* [64,65]. *RUNX2* is also a TGFβ target gene [18,66–68], and RUNX2 is a transcriptional partner for SMADs [41]. An early study shows that activated SMADs are directed to subnuclear foci only in the presence of RUNX proteins, and the SMAD-RUNX complexes are associated in situ with the nuclear matrix. In addition, RUNX2 recruits SMADs to subnuclear sites of active transcription [69]. The 391–432 domain of RUNX2 is required for the interaction of RUNX2 with SMAD3. Thus, the Smad-interacting domain (SMID) in RUNX2 overlaps the nuclear matrix targeting signal [70]. The partnership between RUNX2 and SMADs in promoting EMT and tumor progression is illustrated in human breast and prostate cancer. In response to TGFβ, RUNX2 interacts with SMAD3 and JUNB at the distal runt domain (RD) site of the *MMP13 (collagenase-3)* promoter in MDA-MB-231 breast cancer cells [18]. RUNX2 induces EMT, with increased expression of the EMT-related transcription factor *SNAI2* and decreased expression of *E-cadherin* in breast cancer cells. Inhibition of TGFβ signaling suppresses RUNX2-stimulated *SNAI2* expression [71]. Using a reporter carrying TGFβ responsive motifs flanked by RUNX2 motifs (3x-multimerized TGFβRE-luciferase reporter), luciferase assays show that RUNX2 synergizes with TGFβ/SMADs to increase the promoter activity of the reporter in PC3-a prostate cancer cells [72]. siRNA-mediated RUNX2 depletion impaired the positive transcriptional TGFβ effect on the expression of *cadherin 6* (*CDH6)*, whose product is a potential mesenchymal marker of the EMT program in thyroid cancer, controlling invasiveness of thyroid tumors [73]. Similarly, RUNX2 silencing in human thyroid cancer cell lines results in decreased mRNA expression of *SNAI2* and invasion [74].

In addition to the interdependence of RUNX2 and the TGFβ signaling pathway during the EMT process, the RUNX2-SMADs partnership is central for tumor growth, metastasis and bone disease. RUNX2 is an integral part of TGFβ-mediated *PTHrP* and *cyclin D1* gene regulation in breast cancer cells, facilitating the vicious cycle of cancer cell survival and osteolytic disease [14]. Mutation of three residues in RUNX2 leading to impaired recruitment of SMADs to RUNX2 subnuclear foci reduces prostate tumor size and osteolytic disease in the intratibial model. This study also identifies that *VEGF* and osteolytic related genes such as *osteoprotegerin, PAI-1, PTHrP and IL-11* are under the control of the RUNX2-SMAD interaction [75]. Furthermore, RUNX2-SMAD interaction is crucial for distal metastasis of prostate cancer cells from bone to lung [75].

The mechanisms involved in the cooperation between TGFβ and PI3K/AKT in cancer progression have been reviewed extensively [30,76]. This cooperation takes place in advanced cancer stages to promote EMT, and subsequent migration, invasion and metastasis. This signaling interplay includes a SMAD-independent, rapid induction of AKT phosphorylation by TGFβ and an indirect activation of PI3K/AKT by TGFβ-induced miRNA expression [30]. Reciprocally, PI3K/AKT activation promotes TβRI stability for sustained TGFβ signaling. The ubiquitin-specific protease, USP4 was described as a potent enhancer of TGF-β signaling by directly deubiquitylating the receptor TβRI, thereby maintaining sustained TβRI levels at the plasma membrane. AKT phosphorylates USP4, leading to USP4 relocalization to the membrane, reinforcing the pro-tumorigenic functions of TGFβ [77]. In addition, AKT directly phosphorylates TWIST1 at Ser42 to promote TWIST1-mediated expression of TGFβ2, leading to enhanced TGFβ signaling in breast cancer cells [31]. Furthermore, activation of the PI3K/AKT pathway phosphorylates and inactivates GSK3β, suppressing GSK3β-mediated negative regulation of another EMT factor, SNAIL in breast cancer cells [78].

Therefore, the positive feedback loop between PI3K/AKT and RUNX2 strengthens the collaboration between SMADs and RUNX2 and between PI3K/AKT and TGFβ and participates in the reprogramming of cancer cells to express EMT transcription factors, MMPs, VEGF, bone matrix proteins, bone-resorbing factors, and other products of genes associated with invasion and metastasis. Interestingly, growing evidence shows that the EMT transcription factors regulate not only EMT and tumor invasiveness but also multiple hallmarks of cancer, including angiogenesis, sustained proliferative signaling, evasion from growth inhibitory signals, or resisting cell death by conferring resistance to chemotherapy and radiotherapy [79].

## Contribution of the RAS/MAPK pathway and PI3K/AKT-RAS/MAPK cross-talk to RUNX2 activity

In response to growth factors stimulation, receptor tyrosine kinases (RTKs) and other membrane receptors activate the RAS/MAPK/ERK and PI3K/AKT signaling pathways [80,81]. In the context of cancer cells where autocrine and paracrine activation of growth factor receptors contribute to tumor progression, RUNX2 undergoes phosphorylation by both ERK and AKT kinases. ERK phosphorylation sites in RUNX2 (S43, S301, S309 and S510) have been identified and functionally characterized [82]. The double S301A/S319A phosphorylation site mutation significantly reduces expression of *VEGF, MMP9 and SPP1*, migration and invasion of human prostate cancer cell lines and in vivo growth of tumor cell xenografts [83]. Another study demonstrates that activation of the TAK1-MKK3/6-p38 MAPK axis leads to phosphorylation of RUNX2 by p38, promoting RUNX2 association with the co-activator CREB-binding protein, CBP, which is required to regulate osteoblast genetic programs [84]. Signaling crosstalk represents an important component regulating RUNX2 as a driver of tumor progression via phosphorylation. The phosphorylation of RUNX2 by AKT, ERK and p38 and the effect of these phosphorylation events on RUNX2 activity have to be contextualized in tumor cells exhibiting continuous cross-talk between the RAS/MAPK and PI3K/AKT pathways [80]. In addition to being a downstream target of this crosstalk, RUNX2 also acts on this crosstalk. RUNX2 promotes the crosstalk between MEK/ERK and PI3K/AKT via EGFR in human MCF-10A mammary epithelial cells [85].

## Conclusion

The transcription factor RUNX2 and the PI3K/AKT axis are key players of EMT, migration and invasion. Overexpression of activated RUNX2 and hyperactivation of AKT represent two characteristics of pro-migratory and pro-invasive tumor cells. As shown in non-transformed and transformed cells, the PI3K/AKT pathway directly or indirectly stimulates RUNX2 expression and activity, while RUNX2 activates the PI3K/AKT pathway by the regulation of its components or mTORC2. In the context of cancer cells, this reciprocal activation may set the stage for uncontrolled expression of genes involved in promoting tumor progression (Figs. 1c and 2). However, some important questions remain unanswered: How does AKT participate in the switch of RUNX2 from a tumor suppressor [86] to a pro-oncogenic factor at advanced stages of cancer? What is the role of the RAS/MAPK-PI3K/AKT crosstalk in this switch? In addition to phosphorylating and inhibiting negative regulators of RUNX2, such as FOXO factors, SMURF2 or GSK3β, what interactions between RUNX2 and other transcription factors/co-activators are promoted by direct phosphorylation of RUNX2 by AKT? What metastasis-related genes are induced by these AKT-mediated interactions? Can these AKT-mediated interactions between RUNX2 and other transcription factors/co-activators be targeted as opposed to targeting RUNX2 alone, which remains a challenge? As it appears that two or even the three RUNX proteins are often co-expressed in tumor cells, how does the phosphorylation of RUNX1 and RUNX3 by AKT modulate the effects of AKT-mediated RUNX2 phosphorylation on tumor progression? Are these modulations mediated through direct or indirect interactions between RUNX2 and RUNX1 or RUNX3? Understanding how partnership between transcription factors/co-activators can promote the expression of genes involved in tumor progression might be an indispensable step to overcome the present challenge of targeting pro-oncogenic transcription factors, traditionally considered undruggable.

Although the focus of this review is the PI3K/AKT and RUNX2 mutual activation, the integration of the TGFβ signaling pathway in this positive feedback loop further illustrates the complexity of the interactions governing tumor progression, which warrants a comparable level of complexity for the design of new treatments for cancer patients. Therefore, the identification of such positive feedbacks loops in tumor cells may provide insight into potential new combination treatments. Instead of targeting one player of the metastatic process, inevitably resulting in resistance mechanisms, simultaneous targeting of partners of positive feedbacks loops, such as PI3K/AKT, RUNX2 and TGFβ, may substantially compromise the strategies developed by tumor cells to generate resistance mechanisms.

## References

[1] Blyth K, Vaillant F, Jenkins A, McDonald L, Pringle MA, Huser C, Stein T, Neil J, Cameron ER (2010). Runx2 in normal tissues and cancer cells: A developing story. Blood Cells Mol Dis.

[2] Sun SS, Zhang L, Yang J, Zhou X: Role of runt-related transcription factor 2 in signal network of tumors as an inter-mediator. Cancer Lett. 2015;361:1–7 10.1016/j.canlet.2015.02.042 25727319

[3] Karsenty G, Kronenberg HM, Settembre C (2009). Genetic control of bone formation. Annu Rev Cell Dev Biol.

[4] Provot S, Schipani E (2005). Molecular mechanisms of endochondral bone development. Biochem Biophys Res Commun.

[5] Mackie EJ, Tatarczuch L, Mirams M (2011). The skeleton: a multi-functional complex organ: the growth plate chondrocyte and endochondral ossification. J Endocrinol.

[6] Chen H, Ghori-Javed FY, Rashid H, Adhami MD, Serra R, Gutierrez SE, Javed A (2014). Runx2 regulates endochondral ossification through control of chondrocyte proliferation and differentiation. J Bone Miner Res.

[7] Blyth K, Vaillant F, Hanlon L, Mackay N, Bell M, Jenkins A, Neil JC, Cameron ER (2006). Runx2 and MYC collaborate in lymphoma development by suppressing apoptotic and growth arrest pathways in vivo. Cancer Res.

[8] Colla S, Morandi F, Lazzaretti M, Rizzato R, Lunghi P, Bonomini S, Mancini C, Pedrazzoni M, Crugnola M, Rizzoli V, Giuliani N (2005). Human myeloma cells express the bone regulating gene Runx2/Cbfa1 and produce osteopontin that is involved in angiogenesis in multiple myeloma patients. Leukemia.

[9] Kuo YH, Zaidi SK, Gornostaeva S, Komori T, Stein GS, Castilla LH (2009). Runx2 induces acute myeloid leukemia in cooperation with Cbfbeta-SMMHC in mice. Blood.

[10] Pratap J, Lian JB, Javed A, Barnes GL, van Wijnen AJ, Stein JL, Stein GS (2006). Regulatory roles of Runx2 in metastatic tumor and cancer cell interactions with bone. Cancer Metastasis Rev.

[11] Pratap J, Lian JB, Stein GS (2011). Metastatic bone disease: role of transcription factors and future targets. Bone.

[12] Sun SS, Zhang L, Yang J, Zhou X (2015). Role of runt-related transcription factor 2 in signal network of tumors as an inter-mediator. Cancer Lett.

[13] Akech J, Wixted JJ, Bedard K, van der Deen M, Hussain S, Guise TA, van Wijnen AJ, Stein JL, Languino LR, Altieri DC (2010). Runx2 association with progression of prostate cancer in patients: mechanisms mediating bone osteolysis and osteoblastic metastatic lesions. Oncogene.

[14] Pratap J, Wixted JJ, Gaur T, Zaidi SK, Dobson J, Gokul KD, Hussain S, van Wijnen AJ, Stein JL, Stein GS, Lian JB (2008). Runx2 transcriptional activation of Indian Hedgehog and a downstream bone metastatic pathway in breast cancer cells. Cancer Res.

[15] Baniwal SK, Khalid O, Gabet Y, Shah RR, Purcell DJ, Mav D, Kohn-Gabet AE, Shi Y, Coetzee GA, Frenkel B (2010). Runx2 transcriptome of prostate cancer cells: insights into invasiveness and bone metastasis. Mol Cancer.

[16] Mendoza-Villanueva D, Deng W, Lopez-Camacho C, Shore P (2010). The Runx transcriptional co-activator, CBFbeta, is essential for invasion of breast cancer cells. Mol Cancer.

[17] Pratap J, Javed A, Languino LR, van Wijnen AJ, Stein JL, Stein GS, Lian JB (2005). The Runx2 osteogenic transcription factor regulates matrix metalloproteinase 9 in bone metastatic cancer cells and controls cell invasion. Mol Cell Biol.

[18] Selvamurugan N, Kwok S, Partridge NC (2004). Smad3 interacts with JunB and Cbfa1/Runx2 for transforming growth factor-beta1-stimulated collagenase-3 expression in human breast cancer cells. J Biol Chem.

[19] Pratap J, Imbalzano KM, Underwood JM, Cohet N, Gokul K, Akech J, van Wijnen AJ, Stein JL, Imbalzano AN, Nickerson JA (2009). Ectopic runx2 expression in mammary epithelial cells disrupts formation of normal acini structure: implications for breast cancer progression. Cancer Res.

[20] Owens TW, Rogers RL, Best SA, Ledger A, Mooney AM, Ferguson A, Shore P, Swarbrick A, Ormandy CJ, Simpson PT (2014). Runx2 is a novel regulator of mammary epithelial cell fate in development and breast cancer. Cancer Res.

[21] Sancisi V, Borettini G, Maramotti S, Ragazzi M, Tamagnini I, Nicoli D, Piana S, Ciarrocchi A (2012). Runx2 isoform I controls a panel of proinvasive genes driving aggressiveness of papillary thyroid carcinomas. J Clin Endocrinol Metab.

[22] Sase T, Suzuki T, Miura K, Shiiba K, Sato I, Nakamura Y, Takagi K, Onodera Y, Miki Y, Watanabe M (2012). Runt-related transcription factor 2 in human colon carcinoma: a potent prognostic factor associated with estrogen receptor. Int J Cancer.

[23] van der Deen M, Akech J, Lapointe D, Gupta S, Young DW, Montecino MA, Galindo M, Lian JB, Stein JL, Stein GS, van Wijnen AJ (2012). Genomic promoter occupancy of runt-related transcription factor RUNX2 in Osteosarcoma cells identifies genes involved in cell adhesion and motility. J Biol Chem.

[24] Boregowda RK, Olabisi OO, Abushahba W, Jeong BS, Haenssen KK, Chen W, Chekmareva M, Lasfar A, Foran DJ, Goydos JS, Cohen-Solal KA (2014). RUNX2 is overexpressed in melanoma cells and mediates their migration and invasion. Cancer Lett.

[25] Sun L, Vitolo M, Passaniti A (2001). Runt-related gene 2 in endothelial cells: inducible expression and specific regulation of cell migration and invasion. Cancer Res.

[26] Pierce AD, Anglin IE, Vitolo MI, Mochin MT, Underwood KF, Goldblum SE, Kommineni S, Passaniti A (2012). Glucose-activated RUNX2 phosphorylation promotes endothelial cell proliferation and an angiogenic phenotype. J Cell Biochem.

[27] Kwon TG, Zhao X, Yang Q, Li Y, Ge C, Zhao G, Franceschi RT (2011). Physical and functional interactions between Runx2 and HIF-1alpha induce vascular endothelial growth factor gene expression. J Cell Biochem.

[28] Lee SH, Che X, Jeong JH, Choi JY, Lee YJ, Lee YH, Bae SC, Lee YM (2012). Runx2 Protein Stabilizes Hypoxia-inducible Factor-1alpha through Competition with von Hippel-Lindau Protein (pVHL) and Stimulates Angiogenesis in Growth Plate Hypertrophic Chondrocytes. J Biol Chem.

[29] Bakin AV, Tomlinson AK, Bhowmick NA, Moses HL, Arteaga CL (2000). Phosphatidylinositol 3-kinase function is required for transforming growth factor beta-mediated epithelial to mesenchymal transition and cell migration. J Biol Chem.

[30] Zhang L, Zhou F, ten Dijke P (2013). Signaling interplay between transforming growth factor-beta receptor and PI3K/AKT pathways in cancer. Trends Biochem Sci.

[31] Xue G, Restuccia DF, Lan Q, Hynx D, Dirnhofer S, Hess D, Ruegg C, Hemmings BA (2012). Akt/PKB-mediated phosphorylation of Twist1 promotes tumor metastasis via mediating cross-talk between PI3K/Akt and TGF-beta signaling axes. Cancer Discov.

[32] Kim D, Kim S, Koh H, Yoon SO, Chung AS, Cho KS, Chung J (2001). Akt/PKB promotes cancer cell invasion via increased motility and metalloproteinase production. Faseb J.

[33] Meng Q, Xia C, Fang J, Rojanasakul Y, Jiang BH (2006). Role of PI3K and AKT specific isoforms in ovarian cancer cell migration, invasion and proliferation through the p70S6K1 pathway. Cell Signal.

[34] Chinni SR, Sivalogan S, Dong Z, Filho JC, Deng X, Bonfil RD, Cher ML (2006). CXCL12/CXCR4 signaling activates Akt-1 and MMP-9 expression in prostate cancer cells: the role of bone microenvironment-associated CXCL12. Prostate.

[35] Selvaraj N, Budka JA, Ferris MW, Jerde TJ, Hollenhorst PC (2014). Prostate cancer ETS rearrangements switch a cell migration gene expression program from RAS/ERK to PI3K/AKT regulation. Mol Cancer.

[36] Stewart AL, Mhashilkar AM, Yang XH, Ekmekcioglu S, Saito Y, Sieger K, Schrock R, Onishi E, Swanson X, Mumm JB (2002). PI3 kinase blockade by Ad-PTEN inhibits invasion and induces apoptosis in RGP and metastatic melanoma cells. Mol Med.

[37] Nogueira C, Kim KH, Sung H, Paraiso KH, Dannenberg JH, Bosenberg M, Chin L, Kim M (2010). Cooperative interactions of PTEN deficiency and RAS activation in melanoma metastasis. Oncogene.

[38] Horst B, Gruvberger-Saal SK, Hopkins BD, Bordone L, Yang Y, Chernoff KA, Uzoma I, Schwipper V, Liebau J, Nowak NJ (2009). Gab2-mediated signaling promotes melanoma metastasis. Am J Pathol.

[39] Hou CH, Lin FL, Tong KB, Hou SM, Liu JF (2014). Transforming growth factor alpha promotes osteosarcoma metastasis by ICAM-1 and PI3K/Akt signaling pathway. Biochem Pharmacol.

[40] Chuang LS, Ito K, Ito Y (2013). RUNX family: Regulation and diversification of roles through interacting proteins. Int J Cancer.

[41] Miyazono K, Maeda S, Imamura T (2004). Coordinate regulation of cell growth and differentiation by TGF-beta superfamily and Runx proteins. Oncogene.

[42] Ozaki T, Nakagawara A, Nagase H (2013). RUNX Family Participates in the Regulation of p53-Dependent DNA Damage Response. Int J Genomics.

[43] Su B, Gao L, Baranowski C, Gillard B, Wang J, Ransom R, Ko HK, Gelman IH (2014). A genome-wide RNAi screen identifies FOXO4 as a metastasis-suppressor through counteracting PI3K/AKT signal pathway in prostate cancer. PLoS One.

[44] Byon CH, Javed A, Dai Q, Kappes JC, Clemens TL, Darley-Usmar VM, McDonald JM, Chen Y (2008). Oxidative stress induces vascular calcification through modulation of the osteogenic transcription factor Runx2 by AKT signaling. J Biol Chem.

[45] Kang KA, Kim KC, Bae SC, Hyun JW (2013). Oxidative stress induces proliferation of colorectal cancer cells by inhibiting RUNX3 and activating the Akt signaling pathway. Int J Oncol.

[46] Sosa V, Moline T, Somoza R, Paciucci R, Kondoh H, ME LL (2013). Oxidative stress and cancer: an overview. Ageing Res Rev.

[47] Fujita T, Azuma Y, Fukuyama R, Hattori Y, Yoshida C, Koida M, Ogita K, Komori T (2004). Runx2 induces osteoblast and chondrocyte differentiation and enhances their migration by coupling with PI3K-Akt signaling. J Cell Biol.

[48] Kawamura N, Kugimiya F, Oshima Y, Ohba S, Ikeda T, Saito T, Shinoda Y, Kawasaki Y, Ogata N, Hoshi K (2007). Akt1 in osteoblasts and osteoclasts controls bone remodeling. PLoS One.

[49] Pande S, Browne G, Padmanabhan S, Zaidi SK, Lian JB, van Wijnen AJ, Stein JL, Stein GS (2013). Oncogenic cooperation between PI3K/Akt signaling and transcription factor Runx2 promotes the invasive properties of metastatic breast cancer cells. J Cell Physiol.

[50] Kaneki H, Guo R, Chen D, Yao Z, Schwarz EM, Zhang YE, Boyce BF, Xing L (2006). Tumor necrosis factor promotes Runx2 degradation through up-regulation of Smurf1 and Smurf2 in osteoblasts. J Biol Chem.

[51] Choi YH, Kim YJ, Jeong HM, Jin YH, Yeo CY, Lee KY (2014). Akt enhances Runx2 protein stability by regulating Smurf2 function during osteoblast differentiation. Febs J.

[52] David D, Nair SA, Pillai MR (1835). Smurf E3 ubiquitin ligases at the cross roads of oncogenesis and tumor suppression. Biochim Biophys Acta.

[53] Deng L, Huang L, Sun Y, Heath JM, Wu H, Chen Y (2015). Inhibition of FOXO1/3 Promotes Vascular Calcification. Arterioscler Thromb Vasc Biol.

[54] Yang S, Xu H, Yu S, Cao H, Fan J, Ge C, Fransceschi RT, Dong HH, Xiao G (2011). Foxo1 mediates insulin-like growth factor 1 (IGF1)/insulin regulation of osteocalcin expression by antagonizing Runx2 in osteoblasts. J Biol Chem.

[55] Zhang H, Pan Y, Zheng L, Choe C, Lindgren B, Jensen ED, Westendorf JJ, Cheng L, Huang H (2011). FOXO1 inhibits Runx2 transcriptional activity and prostate cancer cell migration and invasion. Cancer Res.

[56] Zhang Y, Gan B, Liu D, Paik JH (2011). FoxO family members in cancer. Cancer Biol Ther.

[57] Kugimiya F, Kawaguchi H, Ohba S, Kawamura N, Hirata M, Chikuda H, Azuma Y, Woodgett JR, Nakamura K, Chung UI (2007). GSK-3beta controls osteogenesis through regulating Runx2 activity. PLoS One.

[58] Cross DA, Alessi DR, Cohen P, Andjelkovich M, Hemmings BA (1995). Inhibition of glycogen synthase kinase-3 by insulin mediated by protein kinase B. Nature.

[59] Edwards H, Xie C, LaFiura KM, Dombkowski AA, Buck SA, Boerner JL, Taub JW, Matherly LH, Ge Y (2009). RUNX1 regulates phosphoinositide 3-kinase/AKT pathway: role in chemotherapy sensitivity in acute megakaryocytic leukemia. Blood.

[60] Chua CW, Chiu YT, Yuen HF, Chan KW, Man K, Wang X, Ling MT, Wong YC (2009). Suppression of androgen-independent prostate cancer cell aggressiveness by FTY720: validating Runx2 as a potential antimetastatic drug screening platform. Clin Cancer Res.

[61] Tandon M, Chen Z, Pratap J (2014). Runx2 activates PI3K/Akt signaling via mTORC2 regulation in invasive breast cancer cells. Breast Cancer Res.

[62] Sulzmaier FJ, Jean C, Schlaepfer DD (2014). FAK in cancer: mechanistic findings and clinical applications. Nat Rev Cancer.

[63] Moustakas A, Heldin CH (2012). Induction of epithelial-mesenchymal transition by transforming growth factor beta. Semin Cancer Biol.

[64] Derynck R, Muthusamy BP, Saeteurn KY (2014). Signaling pathway cooperation in TGF-beta-induced epithelial-mesenchymal transition. Curr Opin Cell Biol.

[65] Lamouille S, Xu J, Derynck R (2014). Molecular mechanisms of epithelial-mesenchymal transition. Nat Rev Mol Cell Biol.

[66] Lee KS, Hong SH, Bae SC (2002). Both the Smad and p38 MAPK pathways play a crucial role in Runx2 expression following induction by transforming growth factor-beta and bone morphogenetic protein. Oncogene.

[67] Lee KS, Kim HJ, Li QL, Chi XZ, Ueta C, Komori T, Wozney JM, Kim EG, Choi JY, Ryoo HM, Bae SC (2000). Runx2 is a common target of transforming growth factor beta1 and bone morphogenetic protein 2, and cooperation between Runx2 and Smad5 induces osteoblast-specific gene expression in the pluripotent mesenchymal precursor cell line C2C12. Mol Cell Biol.

[68] Mohammad KS, Javelaud D, Fournier PG, Niewolna M, McKenna CR, Peng XH, Duong V, Dunn LK, Mauviel A, Guise TA (2011). TGF-beta-RI kinase inhibitor SD-208 reduces the development and progression of melanoma bone metastases. Cancer Res.

[69] Zaidi SK, Sullivan AJ, van Wijnen AJ, Stein JL, Stein GS, Lian JB (2002). Integration of Runx and Smad regulatory signals at transcriptionally active subnuclear sites. Proc Natl Acad Sci U S A.

[70] Afzal F, Pratap J, Ito K, Ito Y, Stein JL, van Wijnen AJ, Stein GS, Lian JB, Javed A (2005). Smad function and intranuclear targeting share a Runx2 motif required for osteogenic lineage induction and BMP2 responsive transcription. J Cell Physiol.

[71] Chimge NO, Baniwal SK, Little GH, Chen YB, Kahn M, Tripathy D, Borok Z, Frenkel B (2011). Regulation of breast cancer metastasis by Runx2 and estrogen signaling: the role of SNAI2. Breast Cancer Res.

[72] van der Deen M, Akech J, Wang T, FitzGerald TJ, Altieri DC, Languino LR, Lian JB, van Wijnen AJ, Stein JL, Stein GS (2010). The cancer-related Runx2 protein enhances cell growth and responses to androgen and TGFbeta in prostate cancer cells. J Cell Biochem.

[73] Sancisi V, Gandolfi G, Ragazzi M, Nicoli D, Tamagnini I, Piana S, Ciarrocchi A (2013). Cadherin 6 is a new RUNX2 target in TGF-beta signalling pathway. PLoS One.

[74] Niu DF, Kondo T, Nakazawa T, Oishi N, Kawasaki T, Mochizuki K, Yamane T, Katoh R (2012). Transcription factor Runx2 is a regulator of epithelial-mesenchymal transition and invasion in thyroid carcinomas. Lab Invest.

[75] Zhang X, Akech J, Browne G, Russell S, Wixted JJ, Stein JL, Stein GS, Lian JB (2015). Runx2-Smad signaling impacts the progression of tumor-induced bone disease. Int J Cancer.

[76] Assinder SJ, Dong Q, Kovacevic Z, Richardson DR (2009). The TGF-beta, PI3K/Akt and PTEN pathways: established and proposed biochemical integration in prostate cancer. Biochem J.

[77] Zhang L, Zhou F, Drabsch Y, Gao R, Snaar-Jagalska BE, Mickanin C, Huang H, Sheppard KA, Porter JA, Lu CX, ten Dijke P (2012). USP4 is regulated by AKT phosphorylation and directly deubiquitylates TGF-beta type I receptor. Nat Cell Biol.

[78] Zhou BP, Deng J, Xia W, Xu J, Li YM, Gunduz M, Hung MC (2004). Dual regulation of Snail by GSK-3beta-mediated phosphorylation in control of epithelial-mesenchymal transition. Nat Cell Biol.

[79] Sanchez-Tillo E, Liu Y, de Barrios O, Siles L, Fanlo L, Cuatrecasas M, Darling DS, Dean DC, Castells A, Postigo A (2012). EMT-activating transcription factors in cancer: beyond EMT and tumor invasiveness. Cell Mol Life Sci.

[80] Aksamitiene E, Kiyatkin A, Kholodenko BN (2012). Cross-talk between mitogenic Ras/MAPK and survival PI3K/Akt pathways: a fine balance. Biochem Soc Trans.

[81] Lemmon MA, Schlessinger J (2010). Cell signaling by receptor tyrosine kinases. Cell.

[82] Ge C, Xiao G, Jiang D, Yang Q, Hatch NE, Roca H, Franceschi RT (2009). Identification and functional characterization of ERK/MAPK phosphorylation sites in the Runx2 transcription factor. J Biol Chem.

[83] Ge C, Zhao G, Li Y, Li H, Zhao X, Pannone G, Bufo P, Santoro A, Sanguedolce F, Tortorella S, et al: Role of Runx2 phosphorylation in prostate cancer and association with metastatic disease. Oncogene. 2015 doi:10.1038/onc.2015;91. [Epub ahead of print]. 10.1038/onc.2015.91 PMC4603996 25867060

[84] Greenblatt MB, Shim JH, Zou W, Sitara D, Schweitzer M, Hu D, Lotinun S, Sano Y, Baron R, Park JM (2010). The p38 MAPK pathway is essential for skeletogenesis and bone homeostasis in mice. J Clin Invest.

[85] Tandon M, Chen Z, Pratap J (2014). Role of Runx2 in crosstalk between Mek/Erk and PI3K/Akt signaling in MCF-10A cells. J Cell Biochem.

[86] Ito Y, Bae SC, Chuang LS (2015). The RUNX family: developmental regulators in cancer. Nat Rev Cancer.

